# P-691. Comparative Analysis of Respiratory Infections: The Role of Respiratory Panel Testing

**DOI:** 10.1093/ofid/ofaf695.904

**Published:** 2026-01-11

**Authors:** Sherlin M S, Suresh Kumar Dorairajan, Hemanth H

**Affiliations:** Sri venkateswara college of pharmacy, Chittoor, Andhra Pradesh, India; Apollo hospitals,Vanagaram, Chennai, Tamil Nadu, India; Sri venkateswara college of pharmacy, Chittoor, Andhra Pradesh, India

## Abstract

**Background:**

Respiratory tract infections (RTIs) are a major health concern worldwide. Many viruses and bacteria can cause similar symptoms, like fever, cough, and sore throat, making it hard to tell them apart without testing. Respiratory panel tests can detect multiple infections in one go and are especially useful for hospitalized or high-risk patients.

**Fig.1.**
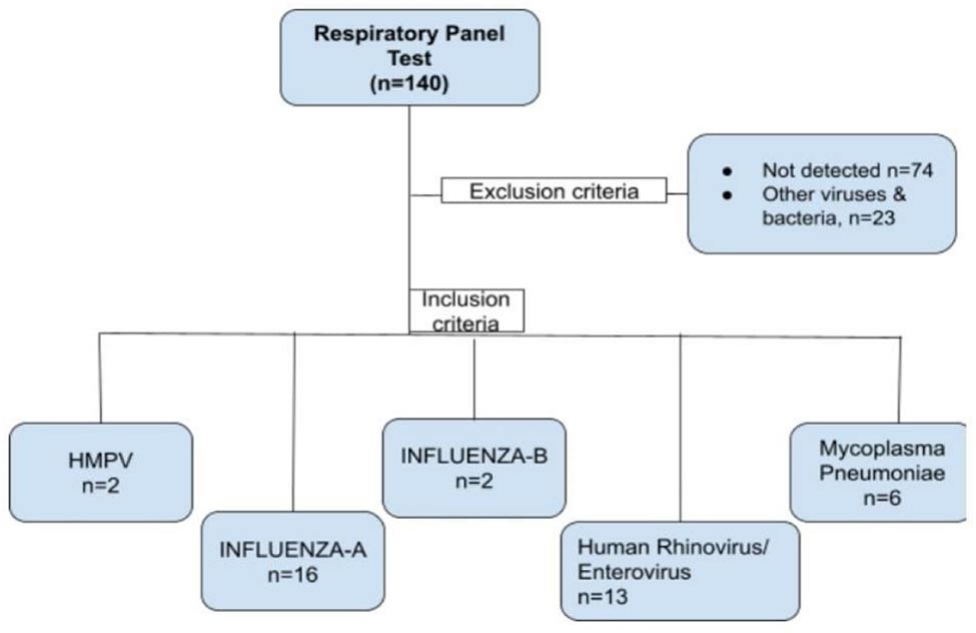
Patient selection

**Figure:**
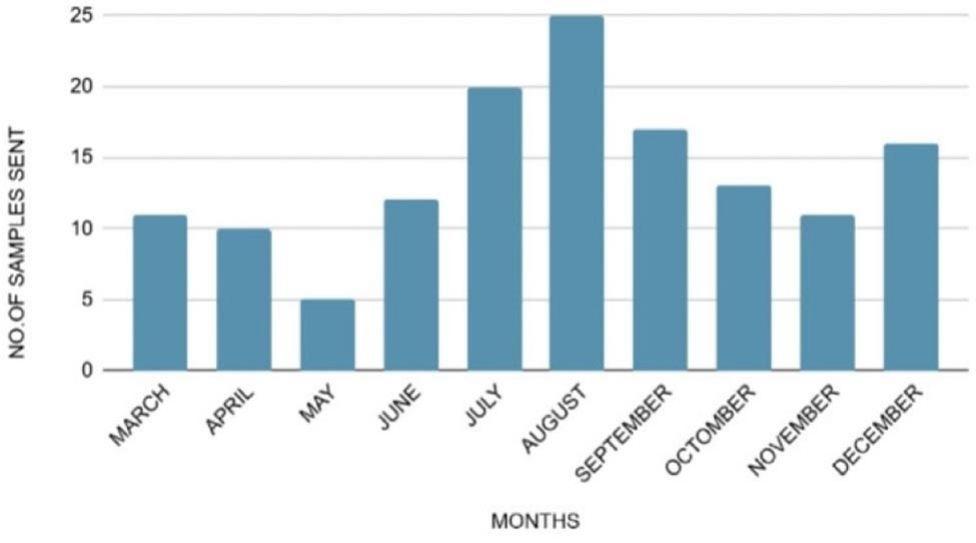
Number of samples sent each month

**Methods:**

This retrospective study was done in a tertiary care hospital in South India from March to December 2024. Medical records of inpatients who underwent respiratory panel testing were reviewed. Common symptoms were compared across viral and bacterial infections.

**Figure:**
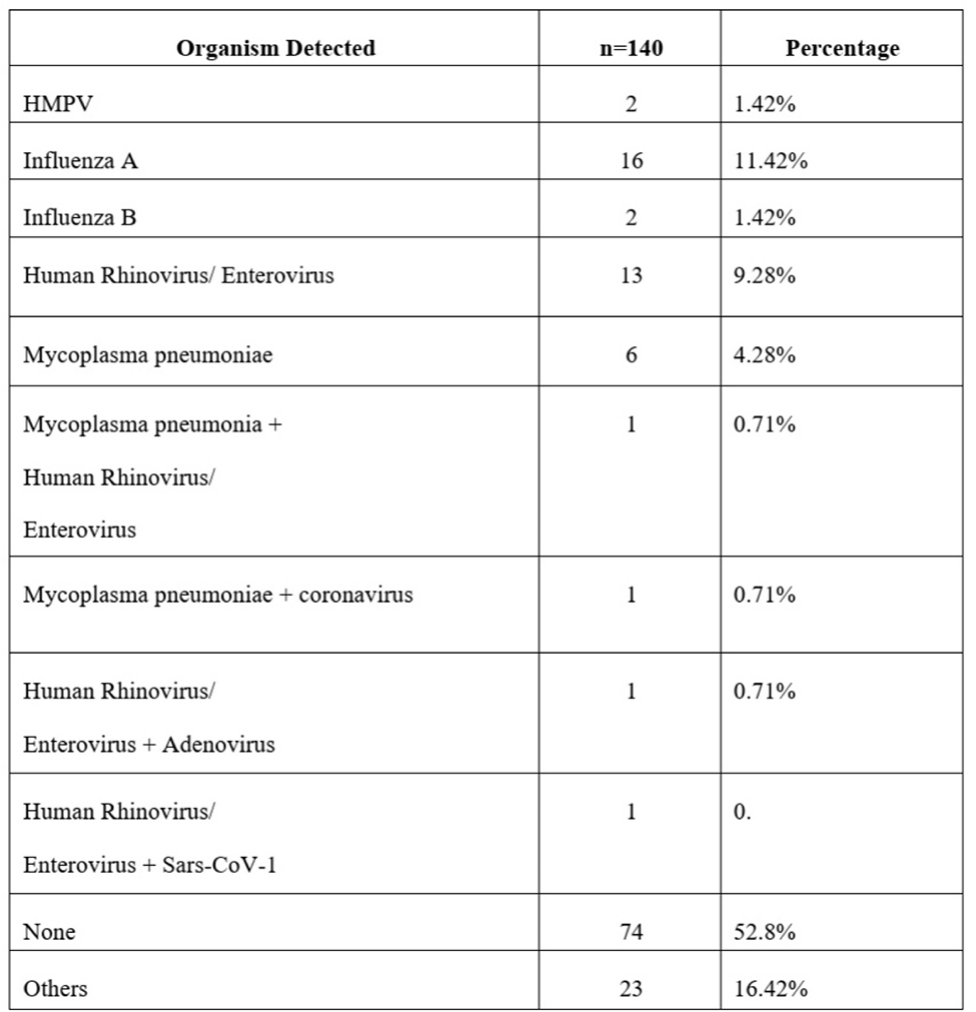
Detected organisms in respiratory samples

**Figure:**
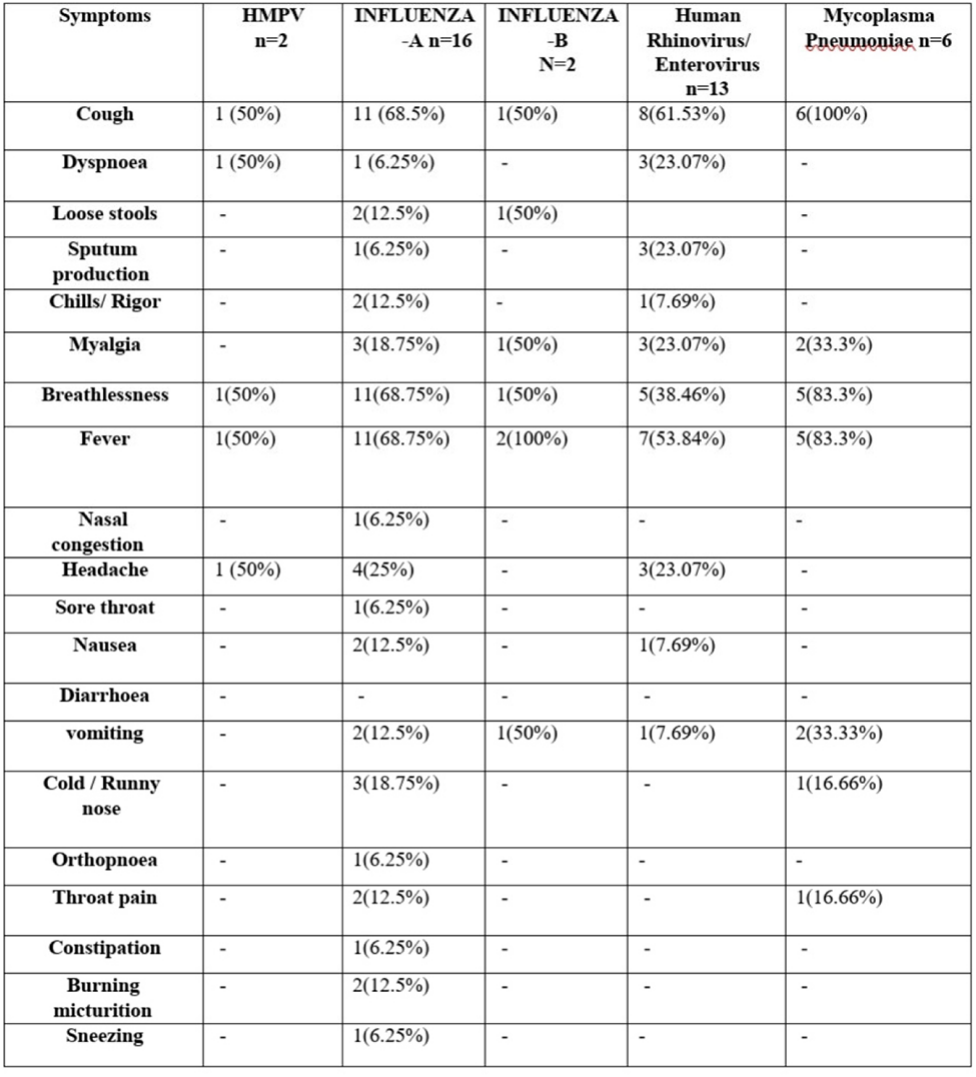
Symptoms associated with different types of respiratory infections

**Results:**

Out of 140 samples [Fig.1], Influenza A was the most common (11.4%), followed by Human Rhinovirus/Enterovirus (9.3%) and Mycoplasma pneumoniae (4.3%). No pathogen was found in 52.8% of cases [Table 1]. Coinfections were rare (0.7%). Most samples were submitted in August [Fig. 2]. Cough was most common in Mycoplasma pneumoniae (100%) and Influenza A (68.5%). Fever and breathlessness were also more common in Mycoplasma cases. Some symptoms, like vomiting and burning urination, were noted in specific infections [Table 2].

**Conclusion:**

Influenza A and Rhinovirus/Enterovirus were the most frequent infections. Mycoplasma pneumoniae caused more severe symptoms. As many respiratory infections present with similar signs, it is difficult to identify the exact cause based on symptoms alone. Therefore, respiratory panel testing is essential for accurate diagnosis, guiding proper treatment, and reducing unnecessary antibiotic use.

**Disclosures:**

All Authors: No reported disclosures

